# Comparing Incommensurable Quantities: Intertemporal vs. Risky Choices with Single Outcomes

**DOI:** 10.3390/bs16030372

**Published:** 2026-03-05

**Authors:** Si-Chu Shen, Yuan-Na Huang, Yi-Juan Zhang, Yi Kuang, Shu-Wen Yang, Shu Li

**Affiliations:** 1 Department of Psychology, Fujian Normal University, Fuzhou 350117, China; 2 School of Management, Jinan University, Guangzhou 510632, China; 3 Department of Psychology, Hubei University, Wuhan 430062, China; 4 Institute of Psychology, Chinese Academy of Sciences, Beijing 100045, China

**Keywords:** intertemporal choices with single-dated outcomes, risky choices with single-nonzero outcomes, comparison between different units of quantity, visual analogue scale, equate-to-differentiate model

## Abstract

The equate-to-differentiate (ETD) model posits that individuals tend to equate a less significant difference between options on one dimension and thus leave the greater one-dimensional difference to be differentiated as the determinant for the preferred option. However, when confronted with an ostensibly “simple” choice between two risky options with single-nonzero outcomes or between two intertemporal options with single-dated outcomes, we face an insurmountable barrier against the ETD model’s explanation and prediction of these choices. The reason is that determining which intra-dimensional difference (∆Payoff*_A,B_* or ∆Probability*_A,B_*/∆Delay*_A,B_*) between Option A and Option B is greater is meaningless and is considered to be a challenge in the physical world. To address this challenge and evaluate whether such decisions are indeed governed by the ETD process, the present study developed a visual analogue scale designed to capture individuals’ subjective comparisons across dimensions of different units. Across two studies, we demonstrate that the analogically measured intra-dimensional comparison reliably and consistently predicts choice patterns attributed to separate anomalies: the common difference effect and unit effect in intertemporal decisions, and subproportionality and the peanuts effect in risky decisions. These findings suggest that both types of decisions may share a common cognitive mechanism based on dimensional evaluation, despite involving distinct informational metrics (time vs. probability). By enabling direct measurement of dimension-wise comparisons, our analogue scale—though unconventional—offers a novel methodological tool for exploring the underlying structure of seemingly “simple” decisions. The implications of this work extend to the development of unified models capable of integrating intertemporal and risky decision-making under a shared explanatory framework.

## 1. Introduction

Intertemporal and risky decision-making are fundamental to understanding human behavior, playing a crucial role in real-world decisions such as saving, health choices, education, and environmental policies. Both types of decisions involve trade-offs: intertemporal choices require evaluating immediate versus delayed rewards, while risky choices involve weighing uncertain outcomes. These decision-making processes share a common structural feature, with outcomes shaped by abstract components like delay or probability, which influence how individuals assess trade-offs between magnitude, uncertainty, and timing. Understanding how individuals make these decisions is vital not only for advancing theoretical frameworks but also for informing practical interventions in real-world decision-making (Frederick et al., 2002).

**Figure 1:**
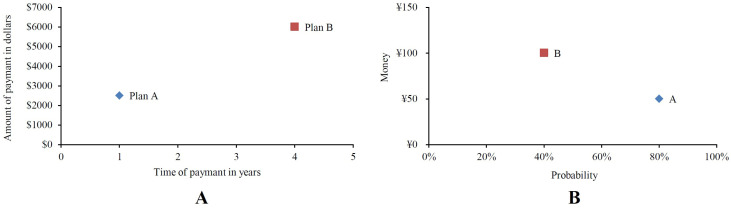
(**A**): Intertemporal choice (i.e., Modified Benefit Problem of Tversky et al., 1988)^1^ between $2500 in one year (Plan A: *SS*) and $6000 in four years (Plan B: *LL*), as similarly illustrated in Li (2004a); (**B**): Risky choice between ¥50 with 80% (A: *P_o_*) and ¥100 with 40% (B: *O_p_*), as similarly illustrated in Sun et al. (2016).

In the domain of intertemporal choices, the most widely studied decisions involve single-dated outcomes. These decisions require choosing between a *smaller but sooner* (*SS*) option and a *larger but later* (*LL*) option (Scholten & Read, 2010). For example, consider the choice between $100 in one month and $150 in four months, shown in Figure 1A. Here, the outcomes are defined as *x_S_* = $100 and *x_L_* = $150, with corresponding times *t_S_* = 1 month and *t_L_* = 4 month.

In the domain of risky choices, the most widely studied choices are those with single-nonzero outcomes (Kahneman & Tversky, 1979), where individuals choose between an option with a *larger outcome and a lower probability* (*O_p_*) and an outcome with a *higher probability and a smaller outcome* (*P_o_*), for instance, choosing between ¥50 with 80% (*P_o_*) and ¥100 with 40% (*O_p_*) (see Figure 1B). In this case, the outcomes are defined as *x* = ¥50 and *y* = ¥100, with probabilities *p* = 80% and *q* = 40%.

The choice between such options represents one of the simplest decision problems, where two options are mapped as unique points in a two-dimensional (2D) choice space (Figure 1). In the case of intertemporal choices, the two dimensions are referred to as the “Amount of Payment” (*x_S_*; *x_L_*) and “Time of Payment” (*t_S_*; *t_L_*) while in risky choices, they are represented by “Amount of Payment” (*x*; *y*) and “Probability of Payment” (*p*; *q*) (or two simple risky dimensions: the probability of winning and the payoff, i.e., Montgomery, 1977).

A key similarity between these decision types is that the binary choice between Option A and Option B is always represented by one “*visible/touchable* dimension” (outcome dimension) and one “*invisible/untouchable* dimension” (probability/time of outcome dimension). Specifically, for intertemporal choices, the visible/touchable dimension is the “Amount of Payment/Outcome” (i.e., *x_S_*; *x_L_*), while the invisible/untouchable dimension is the “Time of Payment” (i.e., *t_S_*; *t_L_*). For risky choices, the visible dimension is the “Amount of Payment/Outcome” (i.e., *x_p_*; *y_q_*), and the invisible dimension is the “Probability of Payment” (i.e., *p_x_*; *q_y_*) (Li, 2004b, p. 159).

In the context of intertemporal utility maximization (c.f., V = Ʃ*d*(*t_i_*)*u*(*x_i_*)) and expectation maximization (c.f., WU = Ʃ*w*(*p_i_*)*u*(*x_i_*)), solving the simplest “single-dated outcome” or “single-nonzero outcome” choice problems is straightforward. These choices primarily involve a weighting or discounting process. In contrast, decisions with “multiple-dated outcomes” (e.g., Scholten et al., 2016) or “multiple outcomes” (e.g., Chateauneuf & Wakker, 1999) require both weighting/discounting and an adding process (e.g., Su et al., 2013).

However, recent studies have challenged the assumption that compensatory and holistic models, which rely on both weighting and summing, provide an adequate explanation for people’s decision-making behaviors. Experimental evidence suggests that these models may not be suitable for accurately modeling risky and intertemporal decisions (e.g., Jiang et al., 2016; H. Z. Liu et al., 2015).

In risky decision-making, L. L. Rao et al. (2012) compared a trade-off instruction choice, requiring participants to integrate probability and payoff, with a preferential choice. fMRI results showed stronger connectivity between the payoff and probability networks during the trade-off task, suggesting that compensatory models’ weighting process is unnecessary for preferential choices. Eye-tracking studies also show that individuals often rely on dimension-based strategies. Su et al. (2013) observed more dimension-based saccades, consistent with non-compensatory models.

In the domain of intertemporal choices, Paul Samuelson’s discounted utility (DU) theory (1937) has been proposed as a normative standard. This model posits that individuals discount future outcomes when making intertemporal decisions. Turner et al. (2019) found that the prefrontal cortex plays a key role in suppressing the impulse for immediate rewards during intertemporal choices, providing support for the discounted utility model. However, numerous anomalies, such as the common difference effect (Kirby & Herrnstein, 1995) and unit effect (Shen et al., 2019), contradict the DU model’s assumptions. To address some of these anomalies, succeeding theories like the hyperbolic discounting model (Mazur, 1984) and quasi-hyperbolic discounting model (Laibson, 1997) were introduced. Yet, new anomalies, such as the intertemporal version of the Allais paradox (L.-L. Rao & Li, 2011), continue to challenge the predictive power of both the DU model and its successors.

Several studies have explored how individuals make intertemporal decisions using *non-compensatory* strategies. For instance, H. Z. Liu et al. (2015) demonstrated that cognitive load and strategy priming influence heuristic processes but not analytic ones, suggesting a non-compensatory priority strategy in intertemporal choices. L. Zhou et al. (2019) used eye-tracking to reveal that both risky and intertemporal choices align with non-compensatory, dimension-based strategies rather than compensatory, holistic models. Collectively, these studies indicate that individuals often rely on simpler, dimension-based strategies rather than complex, weighted computations in decision-making.

In sum, the convergent evidence consistently suggests that risky/intertemporal choices are unlikely to be reached by a weighting/discounting and summing process. In other words, the *visible/touchable* **payoff** is NOT necessarily weighted/discounted by the *invisible/untouchable* **probability**/**time** when making a risky/intertemporal choice.

**Figure 2:**
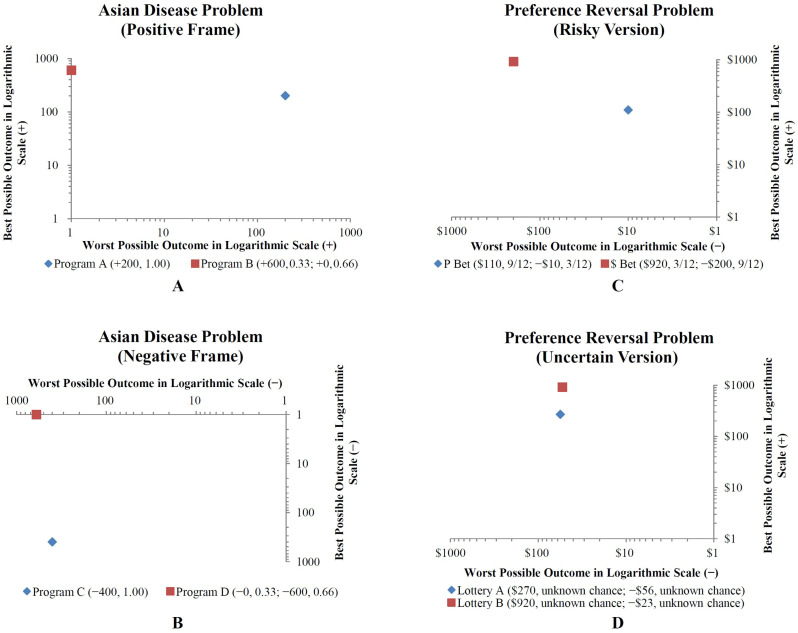
Representation of risky/uncertain choices by applying a logarithmic utility function. (**A**,**B**): risky choice (e.g., Asian disease problem of Tversky & Kahneman, 1981) between a sure option (Program A [+200, 1.00] or Program C [−400, 1.00]) and a risky option with a single-nonzero outcome (Program B [+600, 0.33; + 0, 0.66] or Program D [−0, 0.33; −600, 0.66]), as similarly illustrated in Li (2001, p. 111) or Li and Xie (2006, p. 132). (**C**): risky choice (e.g., the risky version of the preference reversal problem of Lichtenstein & Slovic, 1971) between two risky options with two-nonzero outcomes (*P* Bet [$110, 0.75; −$10, 0.25] vs. *$* Bet [$920, 0.25; −$200, 0.75]), as similarly illustrated in Li (1994, 2016); (**D**): uncertain choice (e.g., uncertain version of the preference reversal problem of Li, 2004b) between two uncertain options with two-nonzero outcomes (Lottery A [$270, unknown chance; −$56, unknown chance] vs. Lottery B [$920, unknown chance; −$23, unknown chance]), as investigated by Li (2004b).

Several researchers have moved away from alternative-based risky and intertemporal choice models, adopting dimension-based models such as the lexicographic semiorder rule (Tversky, 1969), the equate-to-differentiate model (Li, 2004a, 2016), and the tradeoff model (Scholten & Read, 2010). The equate-to-differentiate model posits that individuals seek to equate smaller differences between options on one dimension, leaving the larger difference to determine their choice. In contrast, the lexicographic rule focuses on the most important dimension, and if alternatives are indistinguishable on that dimension, the next most important dimension is considered. Thus, while the lexicographic rule relies on a fixed order of dimensions, the equate-to-differentiate rule allows flexibility, with any dimension serving as the determinant if its difference is perceived as the largest. In the equate-to-differentiate model, risky or uncertain choices can be represented as two points in a 2D choice space (Figure 2). The two dimensions are labeled as the “best possible outcome” and the “worst possible outcome.”

Under the equate-to-differentiate model, two types of risky choices are studied in a 2D choice space. The first type involves a choice between a sure option and a risky option with a single nonzero outcome (Type 1: see Table 1 [1 vs. 2], Figure 2A). The second type involves choosing between two risky options with two nonzero outcomes (Type 2: see Table 1 [2 vs. 2], Figure 2C).

The first type of risky choice (1 vs. 2) involves a sure option (Option A) and a risky option (Option B) with two possible outcomes. The sure option has a single outcome, *x_A_*, with certainty (*p* = 1), while the risky option has two outcomes, *x_B_* (with probability *p_B_*) and *y_B_* (with probability 1—*p_B_*), where *x_B_* > *y_B_*. The decision maker chooses between *x_A_* and *x_B_* if State B occurs, or between *x_A_* and *y_B_* if State W occurs. The best-possible-outcome dimension is represented by *x_B_*, and the worst-possible-outcome dimension by *y_B_*.

The second type of risky choice (2 vs. 2) involves two risky options, each with two possible outcomes. Option A has outcomes *x_A_* (with probability *p_A_*) and *y_A_* (with probability 1—*p_A_*), while Option B has outcomes *x_B_* (with probability *p_B_*) and *y_B_* (with probability 1—*p_B_*), where *x_A_* > *y_A_* and *x_B_* > *y_B_*. The decision maker chooses between *x_A_* and *x_B_* if State B occurs, or between *y_A_* and *y_B_* if State W occurs. The best-possible-outcome dimension is represented by *x_A_* or *x_B_*, and the worst-possible-outcome dimension by *y_A_* or *y_B_*.

The equate-to-differentiate model explains risky choices (1 vs. 2 and 2 vs. 2) by constructing two utility functions over the possible outcomes. The model compares the utility differences between the best possible outcomes (∆ Best possible outcomes) and the worst possible outcomes (∆ Worst possible outcomes) to determine which dimension has the largest difference. Once the dominant dimension is identified, the decision maker’s goal is to select the better outcome along that dimension—either maximizing the best possible outcome or minimizing the worst possible outcome. It is important to note that the best and worst outcomes are commensurable, allowing for direct comparison of their utility differences.

The first application of equate-to-differentiate analysis to intertemporal choice data involved modifying Tversky et al.’s (1988) benefit problem (Li, 2004a; Figure 1, Panel A). This modification allows the intuitive equate-to-differentiate rule to be applied in intertemporal choices.

An attempt was made to analyze L.-L. Rao and Li’s (2011) intertemporal version of the Allais paradox using the equate-to-differentiate model, without proving intra-dimensional evaluation results. The immediacy effect observed in Problem 2 can be interpreted by the equate-to-differentiate rule, where people equate the smaller difference on the “Amount of Payment” dimension (e.g., ¥1,000,000 vs. ¥5,000,000) and leave the larger difference on the “Time of Payment” dimension (e.g., now vs. in 10 years) to determine their final choice (L.-L. Rao & Li, 2011, p. 127).

Ironically, re-examining the existing evidence supporting the equate-to-differentiate model reveals that making an intertemporal choice with a single-dated outcome and a risky choice with a single-nonzero outcome is a deceptively simple yet challenging task. This is because, in non-compensatory and dimensional models, ∆Payoff*_A,B_* represents one unit of quantity, while ∆Probability*_A,B_*/∆Delay*_A,B_* represents another. Comparing ∆Payoff*_A,B_* with ∆Probability*_A,B_*/or ∆Delay*_A,B_* is meaningless, as these dimensions cannot be expressed in the same units. For example, asking whether a kilogram is greater than an hour is nonsensical, as they are incommensurable.

Despite the challenges, several researchers attempt to compare incommensurable quantities. One notable approach is the lexicographic semiorder rule proposed by Tversky (1969), which addresses the trade-off between two incommensurable quantities (e.g., ∆Outcome*_Op,Po_* vs. ∆Probability*_Op,Po_*) in risky choices. This rule works similarly to the lexicographic rule, where the alternative with the best value on the most important dimension is chosen, but with an additional assumption: for the most important dimension, a minimum difference ∆ must exist. If the difference between two options exceeds ∆, the better option on that dimension is selected. If the difference is smaller than or equal to ∆, the decision maker then considers the second most important dimension and selects the option with the higher value on that dimension.

The tradeoff model can be viewed as a refinement of the additive difference model proposed by Tversky (1969). In the tradeoff model, the absolute difference between valued outcomes is weighted against the absolute difference between weighted delays. For a choice between an SS outcome and an LL one, *f* > 0 denotes the time advantage that the SS gain or the LL loss has, and *g* > 0 denotes the outcome advantage that the LL gain or the SS loss has. The decision maker prefers the LL gain or the SS loss if *f* < *g*, prefers the SS gain or the LL loss if *f* > *g*, and is indifferent otherwise (Scholten & Read, 2010).

To capture the more complex and dynamic nature of intertemporal choices, J. Y. Dai and Busemeyer (2014) proposed a probabilistic, dynamic, and attribute-wise model, suggesting that individuals weigh different dimensions, such as outcome magnitude and time delay, when making choices. This study emphasizes the dynamic comparisons between attributes, which is similar to the lexicographic semiorder rule, but applied specifically to intertemporal decision-making. Additionally, Q. Liu (2021) used an eye-tracking technique to reveal how individuals dynamically adjust their attention to different dimensions during decision-making, providing further support for the view that decision-making often involves dimension-based strategies rather than simple compensatory strategies.

Despite using techniques like fMRI (e.g., L. L. Rao et al., 2012), ERP (e.g., L. L. Rao et al., 2013), and eye-tracking (e.g., Su et al., 2013), these studies still cannot directly identify which dimension has the greatest intra-dimensional difference (e.g., ∆Probability_*Op*,*Po*_ or ∆Outcome_*Op*,*Po*_). For example, observed saccades between Payoff*_A_* and Payoff*_B_* or between Probability*_A_* (Delay*_A_*) and Probability*_B_* (Delay*_B_*) can only suggest whether decisions follow an alternative- or dimension-based strategy. However, they do not answer which dimension shows the greatest difference (e.g., ∆Payoff*_A,B_* or ∆Probability*_A,B_*/∆Delay*_A,B_*). Furthermore, Chen and Dai (2025)’s eye-tracking studies show that the information search patterns exhibited by individuals do not always align with their decision strategies in the domain of intertemporal decision-making, suggesting that the issue of which dimension has the greatest difference in the comparison of attribute differences has yet to be resolved using current methods. This lack of intra-dimensional evaluation limits the understanding of decision-making processes in non-compensatory and dimensional models (e.g., lexicographic semiorder rule (Tversky, 1969); equate-to-differentiate model (Li, 2004a); and graph-edited equate-to-differentiate model (Sun et al., 2012)).

The ***visual analogue scale*** was first used in intertemporal choices to determine whether the difference in the time dimension (∆Delay_LL,SS_) is greater than that in the payoff dimension (∆Payoff_LL,SS_) (Jiang et al., 2016). While this scale may not be as natural or convenient as other indicators, such as saccades in eye-tracking experiments, it provides direct evidence of which dimension shows a greater difference and the extent of these differences in a decision maker’s mind. This makes the equate-to-differentiate model, as well as other inter-dimensional models, falsifiable.

This study aims to investigate the predictive validity of the equate-to-differentiate rule using the visual analogue scale. Specifically, Study 1 will test whether knowing which intra-dimensional difference (∆Delay*_A,B_* or ∆Payoff*_A,B_*) between Options A and B is greater can predict an intertemporal choice with a single-dated outcome. Following the same logic, Studies 2 and 3 will apply the visual analogue scale to risky choices with single-nonzero outcomes, examining whether knowing which intra-dimensional difference (∆Probability*_A,B_* or ∆Payoff*_A,B_*) is greater can predict a risk choice.

## 2. Study 1: Understanding the Mechanism of Intertemporal Choice with a Single-Dated Outcome

In a recently published paper, Shen et al. (2019) assessed the replicability of the asymmetric subjective opportunity cost effect (Read et al., 2017) and tested it by setting two additional conditions wherein either LL or SS is more obviously favored (i.e., baseline LL preferences were higher or lower than those in the original condition) by respectively applying the *common difference effect* (Kirby & Herrnstein, 1995) and the *unit effect* (Burson et al., 2009; Pandelaere et al., 2011). The unit effect, which can be viewed as a variant of the magnitude effect (Thaler, 1981), was chosen in this study as it allows for manipulating the outcome dimension through currency units, providing a valuable comparison for understanding how such manipulations influence intertemporal preferences.

The description and prediction of the common difference effect and unit effect appear to be quite different. Nevertheless, the equate-to-differentiate model can provide a simple and coherent explanation of how these two effects can make people’s intertemporal choice shift in two opposite directions (i.e., baseline LL preferences were higher or lower than those in the original condition adopted from Read et al., 2017).

One assumption is that if the difference between two options on the *time* dimension (∆Delay_LL,SS_) is perceived as relatively small, then the objective difference on the time dimension can be subjectively equated by the decision maker, thereby leading to LL being chosen over SS as LL weakly dominates SS (i.e., LL seems as good as SS, having treated the time-dimensional difference as zero, whereas LL is definitely superior than SS on the outcome dimension). Therefore, the equate-to-differentiate way to prompt people to select LL is to make SS as good as LL on the time dimension and then achieve a so-called *equated dominance* (Li & Xie, 2006, p. 131), i.e., LL dominates SS, by treating the smaller time-dimensional difference in which SS is superior to LL as subjectively equal. Coincidentally, applying the *common difference effect* to the original condition (by adding a common constant delay) in Shen et al.’s (2019) replication study is likely to work as expected and prescribed by the equate-to-differentiate model: by making the difference between the SS and LL on the time dimension (∆Delay_LL,SS_) considerably smaller (e.g., in 100 days vs. in 107 days). This approach would make choosing the LL option easy for decision makers who adopt a non-compensatory strategy (e.g., the equate-to-differentiate model).

In the same vein, if the difference between two options on the outcome (i.e., Amount of Payment) dimension (∆Payoff_LL,SS_) is perceived as relatively small, then the objective difference on the outcome dimension can then be subjectively equated by the decision maker, thereby leading to SS being chosen over LL as SS weakly dominates LL (that is, SS seems as good as LL, having treated the outcome-dimensional difference as equal, whereas SS is definitely superior to LL on the time dimension). Correspondingly, in Shen et al.’s (2019) replication study, applying the *unit effect* to the original condition (by replacing the unit of payoffs with the currency of lower value) is likely to make the difference between LL and SS on the outcome (i.e., Amount of Payment) dimension (∆Payoff_LL,SS_) become considerably smaller (e.g., ฿5.40 vs. ฿4.70). This approach would make choosing the SS option easy for equate-to-differentiate practitioners.

Following Li’s (1998) logic^2^ of investigating the framing effect (Tversky & Kahneman, 1981), we reasoned that adding a common constant delay or replacing the unit of payoffs was analogous to the manipulation of framing or wording conditions. If and only if the manipulation of the addition of a common constant delay or replacement of the unit of payoffs could change the perceived relative difference between two options on the time dimension could the so-called common difference effect or unit effect be achieved. That is, the participants’ intertemporal preferences would shift in two opposite directions. Otherwise, the common difference effect or unit effect would not be observed regardless of whether a constant common delay was added or the unit of payoffs was replaced.

Although guided by the equate-to-differentiate model, the common difference effect and the unit effect were successfully replicated and verified in Shen et al.’s (2019) replication study, process evidence has yet to be collected to support the mechanism predicted by the equate-to-differentiate model. Study 1 was therefore designed to examine whether the equate-to-differentiate model can provide a satisfactory account for understanding how the common difference effect and unit effect make people choose between SS and LL options.

### 2.1. Participants

A total of 240 college students (50.4% female; mean age = 21.07 years) were recruited online via Sojump (https://www.wjx.cn/). On the first page of the questionnaire, participants reviewed the study information and provided electronic informed consent before proceeding. After consenting, participants completed an online choice task and received ¥5 as compensation. The participants who failed the attention check were excluded. The study was approved by the Ethical Committee of Fujian Normal University.

### 2.2. Materials and Procedures

The participants were instructed to complete choice and intra-dimensional evaluation tasks. At the start of this study, the participants were instructed to choose between SS and LL options in hypothetical choices. Then, they were asked to perform intra-dimensional evaluation tasks. All these tasks were provided online using the Sojump platform.

***Choice task***: Two pairs of choices, which lead to the *common difference effect*, and two pairs of choices, which lead to the *unit effect*, were taken from Shen et al.’s (2019) replication study. The participants were asked to choose between **SS** and **LL**. The four pairs of choice problems were as follows.

First pair of choices generated the common difference effect (the 6th item was drawn from Magen et al., 2008):

*Without a constant delay added*

A (£4.50, today): smaller but sooner option (*SS*)

B (£7.70, 28 days): larger but later option (*LL*)

*With a constant delay added*

C (£4.50, 100 days): smaller but sooner option (*SS*)

D (£7.70, 128 days): larger but later option (*LL*)

The second pair of choices, generating the common difference effect (the 10th item was drawn from Magen et al., 2008):

*Without a constant delay added*

A (£5.40, today): smaller but sooner option (*SS*)

B (£8.00, 30 days): larger but later option (*LL*)

*With a constant delay added*

C (£5.40, 100 days): smaller but sooner option (*SS*)

D (£8.00, 130 days): larger but later option (*LL*)

The first pair of choices for the unit effect (the 6th item was drawn from Magen et al., 2008):

*Payoff unit of “Pound”*

A (£4.50, today): smaller but sooner option (*SS*)

B (£7.70, 28 days): larger but later option (*LL*)

*Payoff unit of “Thai Baht”*

C (฿4.50, today days): smaller but sooner option (*SS*)

D (฿7.70, 28 days): larger but later option (*LL*)

The second pair of choices for the unit effect (the 10th item was drawn from Magen et al., 2008):

*Payoff unit of “Pound”*

A (£5.40, today): smaller but sooner option (*SS*)

B (£8.00, 30 days): larger but later option (*LL*)

*Payoff unit of “Thai Baht”*

C (฿5.40, today): smaller but sooner option (*SS*)

D (฿8.00, 30 days): larger but later option (*LL*)

In testing the predictions provided by the equate-to-differentiate model, the preference of parallel items should be simultaneously consistent with the prediction produced by the common difference effect and unit effect across different conditions. The criterion of item selection was as follows: compared with the LL preference in the original baseline condition in the work of Read et al. (2017), the proportion of LL choices should be significantly higher in the pair of choices that generated the common difference effect but should be significantly lower in the pair of choices that generated the unit effect. On the basis of the findings of Shen et al.’s (2019) replication study, the 6th and 10th items drawn from Magen et al. (2008) were selected. Moreover, the parallel items, by applying the common difference effect and unit effect, were also included in this work. The two pairs of choice items drawn from Magen et al. (2008) were presented first, followed by the other items, by applying the common difference effect and the unit effect.

**Figure 3:**
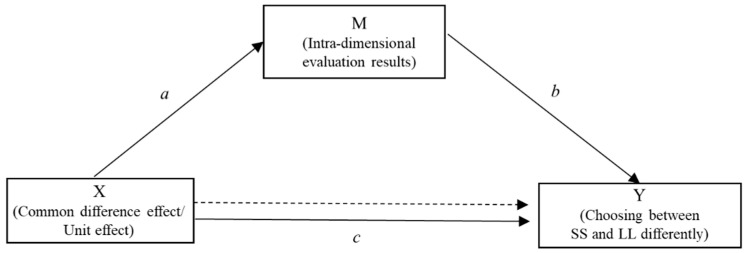
Path diagram form of the mediation model proposed in the present study. a represents the effect of the independent variable X (Common difference effect/Unit effect) on the mediator M (In-tra-dimensional evaluation results); b represents the effect of the mediator M on the dependent variable Y (Choosing between SS and LL differently); c (solid line) represents the total effect of X on Y, while the dashed line represents the direct effect of X on Y after con-trolling for the mediator M.

***Intra-dimensional evaluation task*:** The equate-to-differentiate model, accounting for why the common difference effect and unit effect can make people **choose differently**, can be better understood by viewing the mediator role that intra-dimensional evaluation plays in the following depicted figure (Figure 3). *M* (intra-dimensional evaluation results) mediates the relationship between *X* (by adding a common constant delay/by replacing the unit of payoffs with the currency of lower value) and *Y* (choosing between SS and LL differently). That is, path *a* represents the effect of *X* on mediator *M*, whereas path *b* is the effect of *M* on *Y,* reducing the effect of *X*.

**Figure 4:**
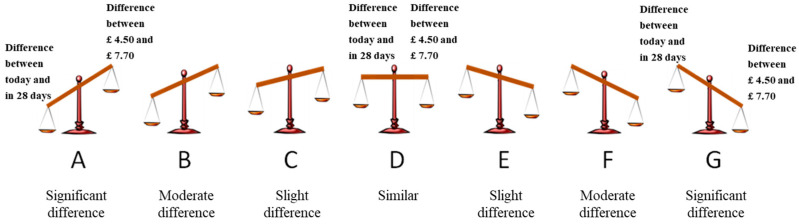
Schematic of the visual analogue scale used in the present study.

To collect evidence that the choice between SS and LL (*Y*) is predicted by the *intra-dimensional evaluation results* (*M*), we need to know which dimension is of relatively greater intra-dimensional difference (∆Payoff*_LL,SS_* or ∆Delay*_LL,SS_*) in the decision maker’s mind and thus serves as the determinant dimension (time or outcome) on which the final decision is based. The visual analogue scale designed and developed by Jiang et al. (2016) can be borrowed and used to help us gain knowledge pertaining to *M* (Figure 4). On the basis of Jiang et al.’s (2016) finding measured by the visual analogue scale, the intra-dimensional evaluation proposed by equate-to-differentiate theory was demonstrated to be a key determinant of people’s choices between SS and LL options. Knowing the determinant dimension, we predicted that the superior option on that dimension would be chosen.

In the visual analogue scale, the difference between two options on the time dimension (∆Delay*_LL,SS_*) was placed on the left side of the scale, whereas the difference between two options on the outcome dimension (∆Payoff*_LL,SS_*) was placed on the right side of the scale. If ∆Delay*_LL,SS_* was larger than ∆Payoff*_LL,SS_*, then the scale would tilt to the left (i.e., A–C). If ∆Payoff*_LL,SS_* was larger than ∆Delay*_LL,SS_*, then the scale would tilt to the right (i.e., E–G). Otherwise, the scale would remain constant when the perceived intra-dimensional difference was nearly equal (i.e., D). The steeper the slope of the scale is, the larger the intra-dimensional difference is in one dimension than in the other dimension. The participants’ task was to rate their subjective evaluation by using a seven-point scale. Take for example the first pairs of choices generating the common difference effect (the 6th item was drawn from Magen et al., 2008). Point A indicated that “the difference between today and in 28 days” was significantly larger than “the difference between £4.5 and £7.7.” Point D indicated that “the difference between today and in 28 days” was equal to “the difference between £4.5 and £7.7.” Point G indicated that “the difference between today and in 28 days” was significantly smaller than “the difference between £4.5 and £7.7.”

Jiang et al.’s (2016) experiments revealed that the order of tasks (first choice task or first intra-dimensional evaluation task) did not have an impact on the mediation role played by the intra-dimensional evaluation results in the choice. For our experiment design, we therefore decided not to balance the effect of the order of presentation to rule out the possibility of the intra-dimensional evaluation being a significant mediator because subjects tried to be consistent between their judgments and their choices.

### 2.3. Results

To test the predicted pattern, we conducted the McNemar test to compare the patience levels of the participants. Then, we further conducted a mediation analysis. Specifically, we investigated whether the perceived intra-dimensional difference results mediated the relationship between applying the common difference effect/the unit effect and the preference change in intertemporal choice. To test the potential mediational relationship formally, we used a bias-corrected bootstrap analysis based on 5000 bootstrap resamples with the SPSS 26.0 macro “MEMORE” provided by Montoya and Hayes (2017) for each choice item. All variables were standardized prior to conducting the mediation analysis.

**Figure 5:**
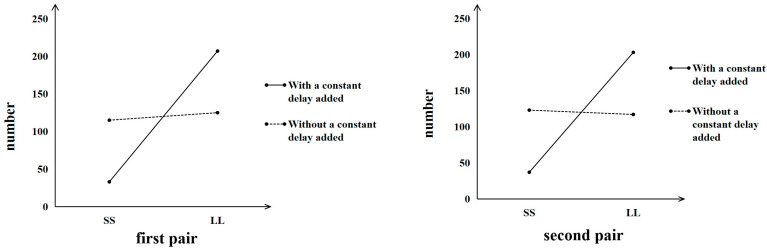
Choice results from the two pairs of choices that generate the common difference effect.

**Figure 6:**
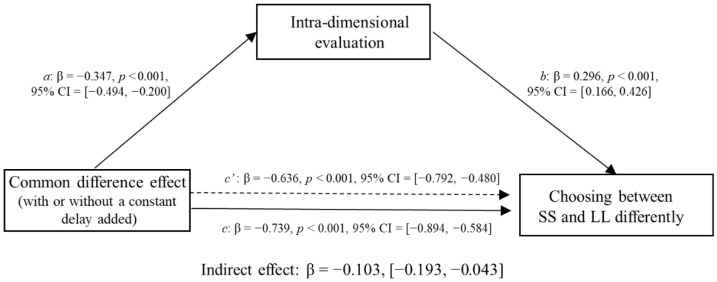
Mediating effects of the common difference effect on choosing between larger but later and smaller but sooner options by the intra-dimensional evaluations of the first pair of choices (the 6th item was drawn from Magen et al., 2008).

**Figure 7:**
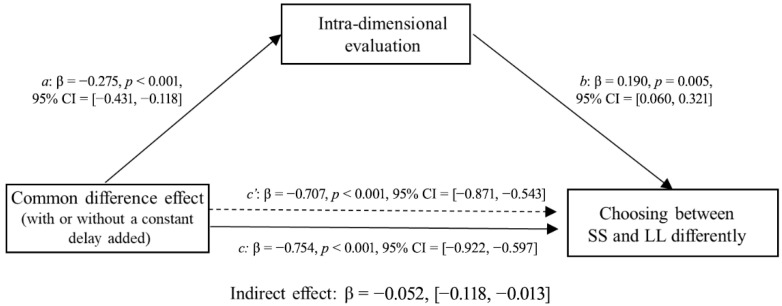
Mediating effects of the common difference effect on choosing between larger but later and smaller but sooner options by the intra-dimensional evaluations of the second pair of choices (the 10th item was drawn from Magen et al., 2008).

First, we examined whether the intra-dimensional evaluations mediated the common difference effect on choosing between the SS and LL options. In this mediation model, the independent variable X (common difference effect) is a binary variable derived from experimental manipulation: the condition without a constant delay was coded as 0, and the condition with a constant delay was coded as 1. The dependent variable Y, representing “Choosing between SS and LL differently”, was calculated as the difference between the number of participants who chose SS and those who chose LL. The mediator variable M was operationalized as the difference in participants’ ratings between the SS and LL options on the visual analogue scale. The McNemar test (Figure 5) showed that the participants were more patient when choosing between options with a constant delay added than when choosing between options without a constant delay added for both items. Specifically, for the first pairs of choices, a larger proportion of participants selected the LL option with the constant delay added, *χ^2^* (1) = 63.09, *p* < 0.001, *phi* = 0.513. On average, 86% of participants chose the LL option when the constant delay was added, compared to 52% choosing LL when no delay was added. A similar pattern was observed for the second pair of choices, with participants more likely to choose the option with the constant delay added, *χ^2^* (1) = 61.23, *p* < 0.001, *phi* = 0.505) (in Shen et al.’s (2019) replication study, the first pairs of choices: *χ^2^* (1) = 348.78, *p* < 0.001, and the second pairs of choices: *χ^2^* (1) = 215.76, *p* < 0.001). The bias-corrected 95% confidence intervals of the indirect effect were [−0.193, −0.043] for the first pairs of choices and [−0.118, −0.013] for the second pairs of choices, neither of which included zero, thereby suggesting that the perceived relative intra-dimensional difference significantly mediated the common difference effect on choosing between the SS and LL options for both choice items (Figure 6 and Figure 7). Adding a common delay made participants perceive smaller relative differences between options on the time dimension than on the outcome dimension; thus, they tended to choose the LL option.

**Figure 8:**
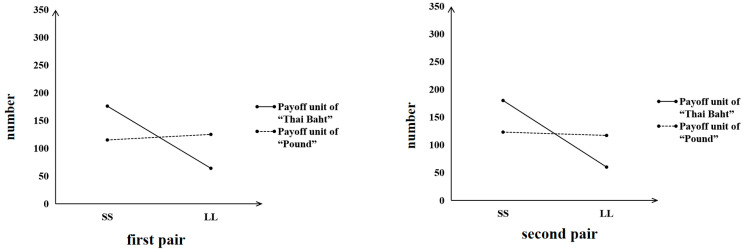
Choice results from the two pairs of choices that generate the unit effect.

**Figure 9:**
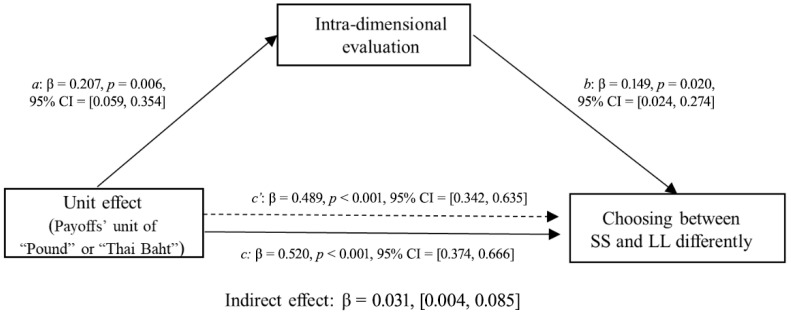
Mediating effects of unit effect on choosing between larger but later and smaller but sooner options by the intra-dimensional evaluations of the first pair of choices (the 6th item was drawn from Magen et al., 2008).

**Figure 10:**
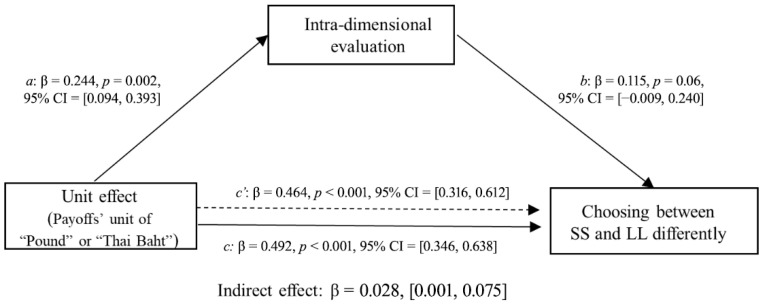
Mediating effects of unit effect on choosing between larger but later and smaller but sooner options by the intra-dimensional evaluations of the second pair of choices (the 10th item was drawn from Magen et al., 2008).

Second, we examined whether the intra-dimensional evaluation results mediated the relationship between applying the unit effect and patience in intertemporal choice. In this mediation model, the independent variable X (unit effect) is a binary variable (payoff unit of ‘pound’ or ‘Thai Baht’) derived from experimental manipulation. The dependent variable Y, representing “Choosing between SS and LL differently,” was calculated as the difference between the number of participants who chose SS and those who chose LL. The mediator variable M was operationalized as the difference in participants’ ratings between the SS and LL options on the visual analogue scale. The McNemar test (Figure 8) showed that the participants were less patient when choosing between options with units of “Thai Baht” than when choosing between options with units of “Pound” for both items. Specifically, for the first pair of choices, participants showed a significant preference for the SS option, *χ^2^* (1) = 39.56, *p* < 0.001. On average, 73% of participants chose the SS option when the payoff unit was “Thai Baht”, compared to 48% choosing SS when the payoff unit was “Pound”. A similar pattern was observed for the second pair of choices, with participants more likely to choose the SS option when the payoff unit was “Thai Baht”, *χ*^2^ (1) = 36.05, *p* < 0.001 (in Shen et al.’s (2019) replication study, the first pair of choices: *χ^2^* (1) = 14.58, *p* < 0.001; the second pair of choices: *χ*^2^ (1) = 20.45, *p* < 0.001). The bias-corrected 95% confidence intervals of the indirect effect were [0.004, 0.085] for the first pair of choices and [0.001, 0.075] for the second pair of choices, neither of which included zero, thereby suggesting that the perceived relative intra-dimensional difference significantly mediated the unit effect on choosing between the SS and LL options for both choice items (Figure 9 and Figure 10). Replacing the unit of payoff made the participants perceive smaller relative differences between options on the outcome dimension than between options on the time dimension; thus, they tended to choose the SS option.

Overall, the findings of Study 1 provided empirical evidence that the common difference effect and unit effect could be satisfactorily accounted for by equate-to-differentiate theory, as revealed by the intra-dimensional evaluation results. Thus, the theoretical account presented in this study can accommodate existing evidence on the mediating effect of intra-dimensional differences on the choice between SS and LL options. Specifically, the participants were more likely to choose the LL option when the perceived relative difference between options on the outcome dimension was larger than that on the time dimension, whereas they were more likely to choose the SS option when the perceived relative difference on the time dimension was larger than that on the outcome dimension.

## 3. Study 2: Understanding the Mechanism of Risky Choice with a Single-Nonzero Outcome: Manipulating the Probability Dimension

Study 1, which investigated intertemporal choice, provided empirical evidence that the *common difference effect* and *unit effect* could be satisfactorily accounted for by equate-to-differentiate theory, as revealed by the intra-dimensional evaluation results. Following the logic of Study 1, Studies 2 and 3 aimed to extend the manipulation of “*invisible/untouchable* dimensional difference” and “*visible/touchable* dimensional difference” to risky choice. In other words, the goal was to manipulate the intra-**probability** dimensional difference (∆Probability_*Op*,*Po*_) and intra-**payoff** dimensional difference (∆Outcome_*Op*,*Po*_) by applying some well-documented effects to risky choice. We seek to explore further whether the equate-to-differentiate model could also provide a satisfactory explanation of how these selected effects can make people’s risky choice shift in two opposite directions.

Given that the attribute/dimension of **time** determines a choice as intertemporal and that the attribute/dimension of **probability** determines a choice as risky, we conducted an experiment (Study 2) in the domain of risky choice by first manipulating the intra-**probability** dimensional difference (∆Probability_*Op*,*Po*_). Considering that subproportionality^3^ (Kahneman & Tversky, 1979) can provide a reasonable explanation for accommodating the famous Allais paradox, we used the subproportionality effect as a representative of manipulating the intra-**probability** dimensional difference (∆Probability_*Op*,*Po*_) in Study 2.

Following the logic of Study 1, we reasoned that if and only if the manipulation of “with *r* multiplied” can change the perceived relative difference between two options on the probability dimension will so-called subproportionality be achieved. That is, the participants’ risky preferences would shift in two opposite directions. Otherwise, subproportionality would not be observed regardless of whether *r* is multiplied.

Accordingly, a set of gamble parameters was deliberately designed to satisfy the choice–shift prediction made by equate-to-differentiate theory, that is, the set of gambling parameters made the perceived relative difference between *P_o_* and *O_p_* on the probability dimension (∆Probability_*Op*,*Po*_) change with *r* multiplied, leading to a prediction made by equate-to-differentiate theory that subproportionality would be observed.

In addition, an improvement was made in Study 2. The indifferent option (option D) in the visual analogue scale that was used in a previous experiment (Figure 4) was excluded to reduce statistical noise.

### 3.1. Participants

A total of 205 college students were recruited online via Sojump (https://www.wjx.cn/). On the first page of the questionnaire, participants reviewed the study information and provided electronic informed consent before participating. They were paid ¥10 each for completing the pair of choice problems that led to the subproportionality. Thirteen participants were excluded owing to their submission of the same responses to all items. Participants who failed the attention check were excluded automatically. Thus, the final valid dataset was 192 (*N*_male_ = 54), and the average age was 20.20 ± 2.12 years. The study was approved by the Ethical Committee of Fujian Normal University.

### 3.2. Procedures and Materials

The participants were asked to perform a choice task first, followed by an intra-dimensional evaluation task.

***Choice task***: The participants were asked to choose between an option with a larger outcome and a lower probability (*O_p_*) and an option with a higher probability and a smaller outcome (*P_o_*). The pair of choice problems led to *subproportionality*, with the following gamble parameters.

*Without r = 0.011 multiplied*

A (¥65,000, 36%): larger outcome with lower probability option (*O_p_*)

B (¥32,500, 72%): higher probability with smaller outcome option (*P_o_*)

*With r = 0.011 multiplied*

C (¥65,000, 0.4%): larger outcome with lower probability option (*O_p_*)

D (¥32,500, 0.8%): higher probability with smaller outcome option (*P_o_*)

**Figure 11:**
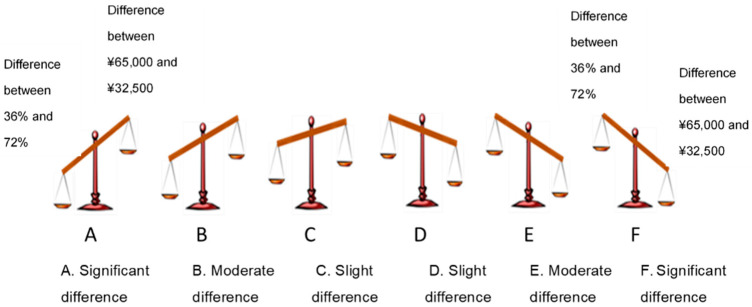
Schematics of the visual analogue scale used in Study 2.

***Intra-dimensional evaluation task***: The participants were asked to rate their subjective intra-dimensional evaluation using a six-point scale. The same instructions used in the previous experiment were utilized (Figure 11).

### 3.3. Results and Analysis

#### 3.3.1. Analysis for Choice Results

**Figure 12:**
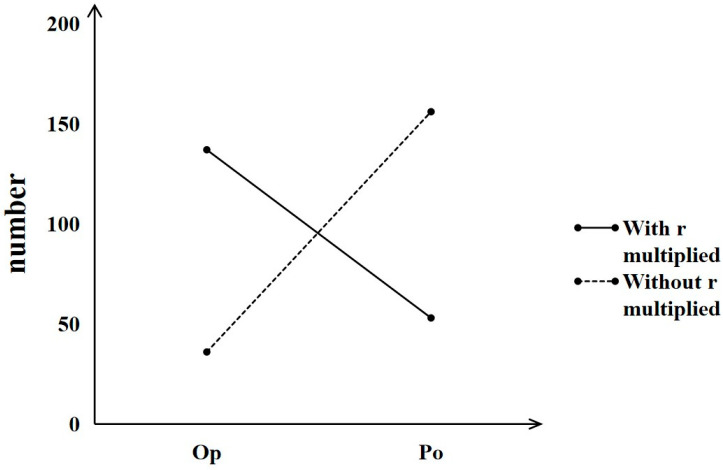
Choice results from the gambling choices that generate subproportionality.

As shown in the results, 81% of the participants in the first pair of choices (without *r* multiplied) chose Option B, which had a higher probability with a smaller outcome (*P_o_*), whereas 72% of the participants chose Option C, which had a larger outcome with a lower probability (*O_p_*) in the second pair of choices (with *r* multiplied) (Figure 12). This finding indicated that the modal preference shifted from Option B to Option C in the expected direction. The difference between the choice proportions in the two pairs of choices was statistically significant, *χ*^2^ (1) = 90.47, *p* < 0.001, *phi* = 0.686, indicating that the majority preference, which generated *subproportionality*, could be replicated successfully by changing the payoff and probability parameters.

#### 3.3.2. Mediation Analysis for Intra-Dimensional Evaluation

**Figure 13:**
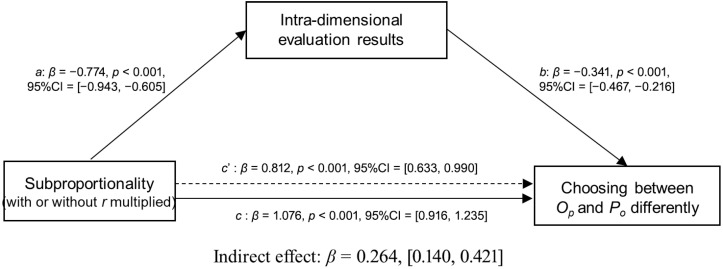
Mediating effects of the subproportionality effect on choosing between the *O_P_* and *P_o_* options through the intra-dimensional evaluation results.

We then examined whether the relationship between subproportionality and choice between the *O_P_* and *P_O_* options can be mediated by the intra-dimensional evaluation results. The independent variable X (subproportionality) is derived from experimental manipulation: the condition without r multiplied was coded as 0, and the condition with r multiplied was coded as 1. The dependent variable Y, representing “Choosing between *O_P_* and *P_o_* differently,” was calculated as the difference between the number of participants who chose *O_P_* and those who chose *P_o_*. The mediator variable M was operationalized as the difference in participants’ ratings between the *O_P_* and *P_o_* options on the visual analogue scale. The data showed that the bias-corrected 95% confidence intervals of the indirect effect were [0.140, 0.421], which do not include zero, suggesting that the perceived intra-dimensional evaluation results significantly mediated the subproportionality in choosing between the *O_P_* and *P_o_* options (Figure 13).

In summary, the findings of Study 2 provided empirical evidence that the subproportionality effect (by manipulating the difference on probability dimension) could be satisfactorily accounted for by equate-to-differentiate theory, as revealed by the intra-dimensional evaluation results.

## 4. Study 3: Understanding the Mechanism of Risky Choices with a Single-Nonzero Outcome: Manipulating the Payoff Dimension

Study 2 presented an attempt to manipulate the intra-**probability** dimensional difference (∆Probability_*Op*,*Po*_) by applying the *subproportionality effect* (Kahneman & Tversky, 1979). Study 3, in turn, aimed to use the *peanuts effect*^4^ (Prelec & Loewenstein, 1991) as a representative of the manipulation of the intra-**payoff** dimensional difference (∆Outcome_*Op*,*Po*_) to determine whether such a manipulation could be satisfactorily accounted for by equate-to-differentiate theory, as revealed by the intra-dimensional evaluation results.

Similar to the previous experiment, the indifferent option (option D) in the visual analogue scale (Figure 4) was excluded to reduce statistical noise.

### 4.1. Participants

A total of 420 participants were recruited online via Sojump (https://www.wjx.cn/) and paid ¥ 7 each for completing the study. On the first page of the questionnaire, participants reviewed the study information and provided electronic informed consent before participating. Nine participants were excluded owing to their submission of the same responses to all items. Participants who failed the attention check were excluded automatically. Thus, the final valid dataset was 411 (*N*_male_ = 141), and the average age was 25.42 ± 6.27 years. The study was approved by the Ethical Committee of Fujian Normal University.

### 4.2. Procedures and Materials

The participants were asked to perform a choice task first, followed by an intra-dimensional evaluation task.

***Choice task***: The participants were asked to choose between a larger outcome with a lower probability option (*O_p_*) and a higher probability with a smaller outcome option (*P_o_*), which might lead to the peanuts effect.

The first pair of choice problems (payoff unit of “US $”) was exactly the one used in Weber and Chapman’s (2005) peanuts effect study.

*Small-stakes gamble*

A ($2, 25%): larger outcome with lower probability option (*O_p_*)

B ($1, 50%): higher probability with smaller outcome option (*P_o_*)

*Large-stakes gamble*

C ($2000, 25%): larger outcome with lower probability option (*O_p_*)

D ($1000, 50%): higher probability with smaller outcome option (*P_o_*)

The second pair of choice problems (payoff unit of “RMB ¥”) was borrowed from the first pair, but its gamble parameters (i.e., “amount of payoff,” “probability of payoff,” and “payoffs’ unit”) were changed completely. The pair is as follows.

*Small-stakes gamble*

A (¥16, 37%): larger outcome with lower probability option (*O_p_*)

B (¥8, 74%): higher probability with smaller outcome option (*P_o_*)

*Large-stakes gamble*

C (¥54,000, 37%): larger outcome with lower probability option (*O_p_*)

D (¥27,000, 74%): higher probability with smaller outcome option (*P_o_*)

***Intra-dimensional evaluation task:*** The participants were then asked to rate their subjective intra-dimensional evaluation using a 6-point scale. The same instructions used in Study 2 were utilized (Figure 11).

### 4.3. Results and Analysis

#### 4.3.1. Analysis for Choice Results

**Figure 14:**
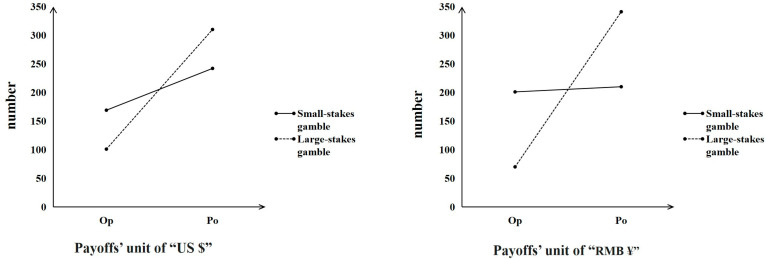
Choice results from the gambling choices that generate the peanuts effect.

Note that the pairs of choices (payoff unit of “US $”) in Study 3 were obtained from Weber and Chapman’s (2005) study, which reported that 59% of the participants chose *O_P_* (A) in a small-stakes gamble and that 84% of the participants chose *P_o_*(D) in a large-stakes gamble. The data of the payoff unit “US $” in Study 3 (Figure 14) showed a somewhat smaller peanuts effect; that is, 41% of the participants chose *O_P_* (A) in a small-stakes gamble, whereas 75% of the participants chose *P_o_*(D) in a large-stakes gamble. However, we did not observe a sharp choice frequency reversal as expected **at the group level** (e.g., from *O_P_* (A), which would be chosen mostly in a small-stakes gamble*,* to *P_o_*(D), which would be chosen frequently in a large-stakes gamble), but worth mentioning is the presence of a significant difference in the choice proportions **at the individual level** (*χ*^2^ (1) = 26.10, *p* < 0.001, *phi* = 0.252), which indicated that the peanuts effect was replicated in the Chinese samples.

On the basis of the experimental evidence reported in Weber and Chapman’s (2005) study, we then explored whether applying the unit of payoff (US $ or RMB ¥) would affect the existence of the peanuts effect. We conjectured that the familiarity with the payoff unit of “RMB ¥” might be higher than the familiarity with the payoff unit of “US $” in our Chinese participants; thus, we used “RMB ¥” as a unit to test whether the peanuts effect can be replicated and enhanced. The present data on the pairs of choices with the payoff unit of “RMB ¥” showed a stronger peanuts effect than the data on the pairs of choices with the payoff unit of “US $” **at the group level**; that is, 49% of the participants chose *O_P_* (A) in a small-stakes gamble, whereas 83% of the participants chose *P_o_*(D) in a large-stakes gamble (Figure 14). The data presented a significant difference between the choice proportions in the two gambles **at the individual level** (*χ*^2^ (1) = 91.35, *p* < 0.001, *phi* = 0.471), again indicating that the peanuts effect was replicated in the Chinese samples.

With respect to the undetected peanuts effect at the group level in Study 3, it can be explained as follows. (a) From the definition of the peanuts effect, we know that when playing for “peanuts,” individuals tend to reduce their risk aversion. It is not actually necessary to become risk-seeking for very small gains, merely to become less risk-averse for smaller payouts. It is thus understandable that the choice proportion for risky choices was chosen under 50% (41% vs. 49%) because the parameters in the small-stakes gamble were considerably small. (b) Weber and Chapman (2005) pointed out that the size of magnitude, the magnitude of the probability, and the ratio between the probabilities of the nonzero payouts in two gambles may influence the strength of the peanuts effect. For instance, when the probability ratio is set to 1.25, the peanuts effect is nonexistent; in fact, for some probability levels, a reverse peanuts effect appears to occur. This evidence indicated that the peanuts effect may not be a common and robust effect. Therefore, it is reasonable to assume that the peanuts effect was replicated and detected in Study 3 even though the choice proportions of *O_p_* (A) in the small-stakes gamble were slightly below 50% **at the group level**.

#### 4.3.2. Mediation Analysis for Intra-Dimensional Evaluation

In this mediation model, the independent variable X (peanuts effect) is a binary variable (small-stakes or large-stakes) derived from experimental manipulation. The dependent variable Y, representing “Choosing between *O_P_* and *P_o_* differently,” was calculated as the difference between the number of participants who chose *O_P_* and those who chose *P_o_*. The mediator variable M was operationalized as the difference in participants’ ratings between the *O_P_* and *P_o_* options on the visual analogue scale.

**Figure 15:**
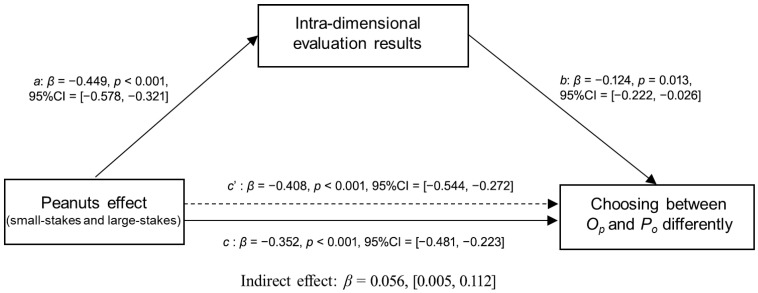
Mediating effects of the peanuts effect on choosing between small-stakes gambles and large-stakes gambles by intra-dimensional evaluations in the payoff unit of **US $ for pairs of choices**.

In the payoff unit of **US $ pairs of choices**, the bias-corrected 95% confidence intervals of the indirect effect were [0.005, 0.112] (Figure 15), which did not include zero, thus suggesting that the perceived relative intra-dimensional difference significantly mediated the relationship between small-large stakes and the choice between *O_P_* and *P_o_.*

**Figure 16:**
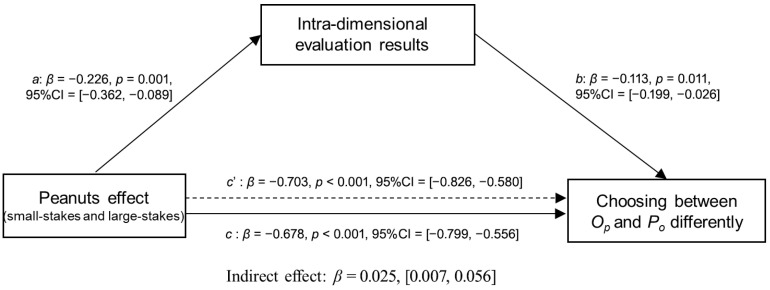
Mediating effects of peanuts effect on choosing between small-stakes and large-stakes gambles by intra-dimensional evaluations of pairs of choices with the payoff unit **“RMB¥”**.

Similarly, in pairs of choices with the payoff unit of “RMB**¥**”, the bias-corrected 95% confidence intervals of the indirect effect were [0.007, 0.056], which did not include zero, indicating that the relationship between small–large stakes and the choice between *O_P_* and *P_o_* can be significantly mediated by the perceived relative intra-dimensional difference (Figure 16).

The findings of Study 3 provided empirical evidence that the peanuts effect (by manipulating the difference in payoff dimension) could be satisfactorily accounted for by equate-to-differentiate theory, as revealed by the intra-dimensional evaluation results.

## 5. Discussion

The present study aimed to investigate how intertemporal choices with a single-dated outcome and risky choices with a single-nonzero outcome can be achieved. While this task may seem straightforward, comparing different units of quantity requires the application of a non-compensatory and dimensional rule, which creates challenges in decision-making. In response to this, we modified the visual analogue scale to examine how intra-dimensional differences can explain intertemporal and risky choices. Specifically, we tested whether the perceived difference in the time (∆Delay) and payoff (∆Payoff) dimensions could account for the common difference effect and unit effect in intertemporal choices, as well as subproportionality and the peanuts effect in risky choices. Our results demonstrate that adjusting the relative differences in the time and outcome dimensions influenced preference for either the LL or SS option in intertemporal choices or the *O_p_* or *P_o_* options in risky choices.

Traditional models of intertemporal and risky decision-making assume compensatory integration of outcomes, such as discounted utility theory (Samuelson, 1937) and expected utility approaches (Kahneman & Tversky, 1979). However, these models often struggle to capture behavioral regularities observed in human choices (e.g., magnitude, immediacy, and probability effects). Recent work using extended model comparisons suggests that attribute comparison frameworks—where risk and time may be treated as independent dimensions—often outperform integrated value approaches in explaining risky and intertemporal choice behavior (L. Dai et al., 2025).

Moreover, process-tracing and gaze-tracking studies have shown that attribute-wise comparison processes often better account for choice dynamics than simple value accumulation models, especially when risky options are judged in parallel across payoff and probability dimensions (Lee et al., 2023). Similarly, eye-tracking and information-search research in intertemporal choice reveals that search patterns do not always map cleanly onto strategy labels, suggesting the need for caution when inferring underlying cognitive processes solely from gaze data (Chen & Dai, 2025).

These findings support the view that decision makers do not always engage in weighted integration across dimensions. Instead, they frequently compare dimensions directly—a pattern that aligns well with non-compensatory and dimensional models like the equate-to-differentiate rule.

In this study, we employed a modified visual analogue scale to overcome the challenge of comparing incommensurable quantities. Prior research has shown that visual and mouse-tracking metrics can independently contribute to predicting choice behavior, reflecting the distributed nature of attribute processing in decision tasks. Unlike many process-tracing approaches that attempt to infer strategy from gaze transitions alone, the visual analogue measure directly captures perceived intra-dimensional differences, providing a more direct test of comparisons hypothesized by non-compensatory models.

However, this method has two key limitations. First, while the visual analogue scale task can directly capture individuals’ relative comparisons across dimensions, its measurement precision remains limited. Specifically, the task primarily focuses on how individuals compare dimensions when presented with different options, but it struggles to comprehensively quantify various factors influencing intertemporal decision-making, such as individuals’ temporal orientation, variations in reward sensitivity linked to subjective socioeconomic status, and impulsivity traits. These factors may introduce “noise” into the dimensional comparison process (Kahneman et al., 2021), which could contribute to residual effects between X and Y. Additionally, while our study finds that the unit effect can be explained by the equate-to-differentiate (ETD) theory and effectively alters individuals’ preferences, we must also acknowledge the presence of other potential confounds. These include individual familiarity with and preference for specific currencies, as well as trust in the countries issuing those currencies and perceptions of inflation rates. These factors may influence the purity of the results of comparisons measured by the visual analogue scale. Second, in intertemporal decision-making, individuals not only must trade off time and money but also must consider multiple dimensions, such as risk perception and future expectations, which are challenging to capture accurately through relative dimensional comparisons alone. Therefore, while the visual analogue scale task effectively captures individuals’ relative preferences at the time of choice, it does not fully reflect the complexity of the entire trade-off process that individuals undergo in decision-making.

Our findings reveal that manipulating perceived differences along the time and payoff dimensions significantly influenced preferences. In intertemporal choice, biasing the time difference increased preference for LL options, whereas biasing the payoff difference favored SS options. This pattern dovetails with the literature showing contextual and task effects on preference reversals in intertemporal choice (Y. B. Zhou et al., 2024), underscoring the importance of domain-specific comparisons in explaining choice behavior.

Likewise, in the risky domain, shifts in relative differences in probability and payoff influenced risk preferences in predictable ways. These patterns align with research emphasizing the role of attribute salience and independent attribute processing in risky choice (Lee et al., 2023). The findings from this study offer a theoretical contribution by extending the equate-to-differentiate rule to explain decisions that involve comparisons of incommensurable quantities. While traditional models such as the discounted utility model rely on compensatory processes, this study suggests that people may rely on non-compensatory, dimensional strategies when making intertemporal and risky choices. Our study highlights the importance of comparing dimensions directly (e.g., time vs. payoff) rather than simply summing values across dimensions.

This decision-making mechanism proposed by the equate-to-differentiate model, which compares differences across dimensions, explains the four effects examined in this study and may also be applicable to other relevant domains. For example, in spatial decision-making, the ETD theory explains the “spatiotemporal framing effect,” where the same decision problem being framed temporally (e.g., “30 min by bus”) versus spatially (e.g., “8 km”) leads to preference reversals. This occurs because the frames emphasize different dimensions, and individuals base their choices on the most prominent differences (Kuang et al., 2023). Similarly, in sustainability-related risk decisions, different formats (e.g., probability per unit time vs. mean time to occurrence) lead to distinct preferences. When the probability is low, presenting risk in terms of mean time to occurrence results in more risk-averse decisions, while high probability leads to more risk-seeking choices. This effect is mediated by judgments of dimensional differences (Wei et al., 2023).

This study contributes to behavioral decision theory by extending the equate-to-differentiate model to account for decisions involving the comparison of incommensurable quantities. Unlike traditional compensatory models, our findings highlight the role of non-compensatory, dimensional strategies in intertemporal and risky choices. By using the visual analogue scale to directly measure intra-dimensional differences, we provide new insights into the mechanisms underlying well-established behavioral effects. These results challenge the conventional view that people make decisions by simple weighting or discounting and instead show that direct dimension comparisons can be predictive of choice behavior.

The findings of this study support the adoption of heuristic strategies based on bounded rationality (Tversky & Kahneman, 1974) in intertemporal and risky decision-making, where individuals simplify information processing to make decisions, rather than adhering to the assumptions of the fully rational economic agent. The rational economic agent model posits that individuals compute the value of each option through weighted or discounted summation during the decision-making process. However, individuals’ choices do not always follow the theoretical predictions of the rational agent model. Instead, anomalies such as the common difference effect/unit effect and subproportionality/peanuts effect, as discussed in this paper, emerge. The findings of this study indicate that individuals do not perform complex calculations on the value of options during decision-making. Rather, they tend to adopt a dimension-based comparison strategy, selecting the option with the higher value on the dimension with the larger difference, while neglecting smaller value differences on the other dimension. This reflects a comparative strategy grounded in bounded rationality. This finding not only supports the theory of bounded rationality (Simon, 1955) in intertemporal and risky decision-making but also provides new empirical evidence for non-compensatory decision strategies based on dimension comparisons, further enriching the theory of heuristic strategies in decision-making processes.

Our study also has important practical implications, especially in the context of decision interventions and nudge theory. By modifying perceived relative differences between options, this research provides a framework for nudging individuals toward more desirable choices—promoting patience in intertemporal decisions or adjusting risk propensities in risky contexts.

Future work could parameterize intra-dimensional differences and integrate them into formal choice models, assessing predictive accuracy against data from both behavioral and process measures (e.g., eye and mouse tracking). Such endeavors would further clarify how attribute comparisons unfold over time and contribute to decision outcomes across domains.

## Figures and Tables

**Table 1:** Organized classification of choice situations (risky and intertemporal) by crossing two factors: types of choice model (compensatory and holistic vs. non-compensatory and dimensional) vs. numbers of outcome (single vs. two vs. multiple).

	Risky Choice			Intertemporal Choice			
	**Single-Nonzero Outcomes**	**Two Outcomes**	**Multiple Outcomes**		**Single-Dated Outcomes**	**Two-Dated Outcomes**	**Multiple-Dated Outcomes**
**compensatory models** *(∑w(p_i_)u(x_i_)))*	*w(p)u(x)*	*w(p)u(x)+w(q)u(y)*	*w(p)u(x)+w(q)u(y)+...*	**compensatory models** *(∑u(x_i_)d(t_i_))*	*u(x)d(t))*	*u(x)d(t_1_))+u(y)d(t_2_))*	*u(x)d(t_1_))+u(y)d(t_2_))+...*
**non-compensatory models** ∆Probability *Op, Po* ∆outcome *Op, Po*	∆Probability*_A,B_* ^1^ and ∆Outcome*_A,B_*	(1vs2). ∆BO*_A,B_* ^2^ and ∆WO*_A,B_* ^2^	unexplored	**non-compensatory models** ∆Delay *LL, SS* ∆Payoff *LL, SS*	∆Delay*_A,B_* ^4^ and ∆Payoff*_A,B_*	∆BO*_A,B_* and ∆WO*_A,B_* ^5^	unexplored
		(2vs2). ∆BO*_A,B_* and ∆WO*_A,B_* ^3^					

^1^ A = *Op* (lager outcome with lower probability option); B = *P_O_* (higher outcome with smaller probability option); ^2^ ∆BO*_A,B_* = relative difference between options on the best-possible-outcome dimension; ∆WO*_A,B_* = relative difference between options on the worst-possible-outcome dimension; ^3^ A = Risky option with two possible outcomes; B = Risky option with two possible outcomes; ^4^ A = *LL*(larger but later option); B = *SS*(smaller but sooner option); ^5^ A = Intertemporal option with two-dated outcomes; B = Intertemporal option with two-dated outcomes.

## Data Availability

The data that support the findings of this study are available from the corresponding authors upon request.

## Notes

1 The outcomes of the two options on the “Time of Payment” dimension are paired as “payment in one year” vs. “payment in four years,” whereas the outcomes of the two options on the “Amount of Payment” dimension are paired as “payment of $2500” vs. “payment of $6000” (Li, 2004a, p. 153).

2 Li (1998) suggested that the framing effect can be produced if and only if framing or wording can change people’s equate-to-differentiate strategy across different frame conditions (see Figure 2A,B), but not otherwise (p. 133).

3 For any fixed probability ratio *r*, the ratio of decision weights is closer to unity when the probabilities are low than when they are high (Kahneman & Tversky, 1979).

4 The effect of decreasing risk aversion with decreasing monetary amounts was christened the “peanuts effect” (Prelec & Loewenstein, 1991), in which decision makers are more willing to take risks when playing for “peanuts.” (By this definition, it is not actually necessary to become risk-seeking for very small gains, merely to become less risk-averse for smaller payouts.)
